# FOOD INSECURITY AMONG OLDER ADULTS IN NEW YORK CITY: DOES LOCATION MATTER?

**DOI:** 10.1093/geroni/igac059.1990

**Published:** 2022-12-20

**Authors:** Ethan Siu Leung Cheung

**Affiliations:** Columbia University, New York, New York, United States

## Abstract

Having access to adequate and appropriate food sources is essential to addressing food insecurity among older adults. However, the role of locational characteristics in explaining food insecurity remains unclear, especially in urban areas. This study investigated the association of distance to grocery stores, neighborhood disadvantage, and social cohesion with food insecurity among older adults in New York City. Individual-level data were drawn from a 2-year Poverty Tracker Study. The sample included New York City residents aged 65 or older (baseline N = 710). Based on the respondents’ residential address and neighborhood ZIP codes, the individual-level data were merged with two spatial datasets: American Community Survey and ReferenceUSA. ArcGIS 10 (near analysis) was used to manage spatial data and calculate the distance to grocery stores. Hierarchical logistic regression models were employed for analyses. Descriptive results show that more older adults in neighborhoods with economic disadvantage and lower level of social cohesion reported more food insecurity. Logistic regressions suggested that after controlling for individual-level characteristics (e.g., age, gender, race and ethnicity, and education), living farther (0.26–0.50 miles and 0.51–0.70 miles) from the nearest grocery store was positively associated with food insecurity. Residing in economically disadvantaged neighborhoods also increased the odds of food insecurity. Community social cohesion was a marginally significant protective factor against food insecurity. Findings suggest that locational characteristics play a significant role in predicting food insecurity in New York City, suggesting that community outreach and grocery delivery programs are needed to mitigate the risk.

